# Downregulation of B3GNT6 is a predictor of poor outcomes in patients with colorectal cancer

**DOI:** 10.1186/s12957-022-02561-x

**Published:** 2022-04-07

**Authors:** Shihan Xiao, Chen Yang, Yang Zhang, Chen Lai

**Affiliations:** 1 Department of General Surgery, Xiangya Hospital of Central South University, Changsha, 410000 Hunan Province China; 2 Hunan Key Laboratory of Precise Diagnosis and Treatment of Gastrointestinal Tumor, Xiangya Hospital of Central South University, Changsha, 410000 Hunan Province China; 3 International Joint Research Center of Minimally Invasive Endoscopic Technology Equipment & Standardization, Xiangya Hospital of Central South University, Changsha, 410000 Hunan Province China; 4 Department of Colorectal Surgery, 1st Affiliated Hospital of Zhejiang University, Hangzhou, 310000 Zhejiang Province China

**Keywords:** B3GNT6, Bioinformatics analysis, TCGA, GSEA, Proteasome

## Abstract

**Background:**

The B3GNT6 protein is a member of the O-GlcNAc transferase (OGT) family and is responsible for the production of the core 3 structure of O-glycans. It is generally expressed in the gastrointestinal (GI) tract; however, its clinical significance in colorectal cancer remains largely unexplored.

**Methods:**

We obtained mRNA transcriptomic sequencing data from 3 gene expression omnibus (GEO) datasets (GSE37182, GSE39582, GSE103512) and The Cancer Genome Atlas (TCGA) to compare the B3GNT6 mRNA levels between colorectal cancer and normal tissues and further evaluate its value as a prognostic marker in colorectal cancer. We further validated this at the protein level in our cohort using immunohistochemical staining of B3GNT6 as well as the Human Protein Atlas online database.

**Results:**

B3GNT6 expression was downregulated in colorectal cancer tissues as compared to that in the normal tissues at both mRNA and protein levels. Downregulation of B3GNT6 expression was found to be associated with poor overall survival in patients with colorectal cancer as per the data in GSE39582 and TCGA databases. Low B3GNT6 mRNA levels were significantly associated with chromosome instability (CIN) and KRAS mutations in patients with colorectal cancer. Gene set enrichment analysis (GSEA) revealed that low B3GNT6 expression levels in colorectal cancer were associated with increased proteasome activity.

**Conclusions:**

The results of this study demonstrate that low expression of B3GNT6 is a potential biomarker for poor outcomes in patients with CRC. Moreover, the low expression of B3GNT6 may indicate more frequent activation of the KRAS/ERK signaling pathway, high CIN, and increased proteasomal activity. These novel findings may prove helpful for molecular diagnosis and provide a new therapeutic target for colorectal cancer.

**Supplementary Information:**

The online version contains supplementary material available at 10.1186/s12957-022-02561-x.

## Introduction

Colorectal cancer accounts for nearly 900,000 deaths annually and is ranked as the world’s 4th most deadly malignancy [1]. Though recent advances in treatment options have almost doubled the overall survival in patients with advanced disease to 3 years, the prognosis is still the best for those with non-metastasized disease [2]. Thus, it is imperative to identify novel prognostic biomarkers for patients with colorectal cancer.

Colorectal cancer is known to comprise various types of genomic mutations that ultimately lead to different prognoses. Chromosome instability (CIN), microsatellite instability (MIN), and CpG island methylator phenotype (CIMP) pathways are the three major well-known molecular pathways in colorectal cancer; each pathway entails varying histology, risk factors, prognosis, and response to therapy [3]. The gene mutations associated with these molecular pathways largely affect patient response to therapy. Herein, prognostic and predictive biomarkers may enable proficient management of colorectal cancer.

The B3GNT6 protein, also known as core 3 synthase, is a member of the O-GlcNAc transferase (OGT) family and adds an *N*-acetylglucosamine to *N*-acetylgalactosamine-modified serine or threonine. The B3GNT6 protein is responsible for the formation of the core 3 structure of O-glycans, which are important components of mucin-type glycoproteins [4]; this process is known as the protein O-GlcNAcylation modification. O-glycans are often mutated or dysregulated in structure in various types of cancers and greatly contribute to the abnormal biological activities seen in cancer cells [5]. Numerous studies have reported that aberrant protein O-GlcNAcylation plays a key role in the growth, metastasis, and progression of many malignant tumors [6–9]. The B3GNT protein family is commonly dysregulated in various types of cancers, including gastrointestinal tumors, cervical cancer, and prostate cancer [9, 10]. B3GNT6 expression has been reported to be largely downregulated in gastric and colorectal cancers [10]. However, the underlying mechanism and relationship between B3GNT6 expression and cancer progression and metastasis remain largely unexplored.

The ubiquitin-proteasome system (UPS) plays a pivotal role in the growth and survival of cancer cells. It is a major component of the cellular protein degradation machinery, which allows the degradation of misfolded and dysregulated proteins in the cells. As an important regulator of a variety of protein substrates, the proteasome contributes virtually to every cellular function, including proliferation, apoptosis, angiogenesis, and metastasis [11]. The proper function of the cellular proteasome is crucial for the survival of both normal and neoplastic cells, especially when the apoptosis proteasome is inhibited. Understanding the function and control of the ubiquitin-proteasome system will further illustrate the regulation of cellular activity and tumor progression.

Herein, we conducted an in-depth bioinformatics analysis using online databases to investigate the mRNA and protein expression patterns of B3GNT6 and its clinical significance in colorectal cancer. Immunohistochemical analysis of B3GNT6 protein expression was also conducted in 43 paired tissues from our own cohort of colorectal cancer patients.

Our results demonstrated that B3GNT6 was downregulated in colorectal cancer tissues as compared with in normal tissues at both mRNA and protein levels. B3GNT6 mRNA expression levels were negatively correlated with overall survival in patients with colorectal cancer. B3GNT6 levels in colorectal cancer patients were associated with CIN and KRAS mutation status. Bioinformatic analyses revealed that downregulation of B3GNT6 expression is enriched in the proteasome pathway.

## Materials and methods

### Data collection and processing

Three datasets including gene expression data of colorectal cancer patients were downloaded from the Gene Expression Omnibus (GEO) database (https://www.ncbi.nlm.nih.gov/geo/). Datasets GSE39582 [12], GSE103582 [13], and GSE37182 [14] contained transcriptomic mRNA expression profiles of 566, 50, and 88 colorectal cancer cases with 19, 84, and 10 normal cases, respectively. Level 3 HTSeq-FPKM files, comprising 612 transcriptome profiling RNA-Seqs of 544 cases, were collected from a dataset in TCGA (https://www.portal.gdc.cancer.gov/) with information on 452 and 96 patients with colon and rectal cancers, respectively. The clinicopathological characteristics, such as age, sex, clinical TNM stage, CIN, MMR, KRAS, and BRAF mutation status were included. The expression levels of the B3GNT6 gene in other cell lines, organs, and cancers were obtained from the MediSapiens IST online database (http://ist.medisapiens.com/). The protein expression levels of B3GNT6 were reviewed using immunohistochemical-staining data provided in the Human Protein Atlas (http://www.proteinatlas.org/).

### Gene set enrichment analysis (GSEA)

To determine the function of B3GNT6, we conducted GSEA using samples with top 25% and bottom 25% of B3GNT6 expression in the GSE39582 dataset with the GSEA 4.1.0 software (https://www.gsea-msigdb.org/gsea). The annotated gene sets c2.cp.kegg.v6.0.symbols.gmt and c2.cp.biocarta.v6.0.symbols.gmt from the pathway database were used as the reference gene sets. The cutoff criteria used were as follows: p < 0.05, |enrichment score (ES)| > 0.3, and gene size ≥ 30.

### Immunohistochemical staining to detect B3GNT6 protein expression in colorectal cancer patients

We obtained 43 cancer tissues, paired adjacent non-tumor tissues, and related clinical information from patients who underwent radical colorectal resection in the Department of General Surgery, Xiangya Hospital of Central South University, between February 2016 and July 2018. All tissues collected were clinically and pathologically diagnosed as colorectal cancer. Patients with recurrence or those who had received adjuvant chemo- or radiotherapy were excluded from our study. The differential expression levels of B3GNT6 protein in 43 colorectal cancer and paired normal tissues were measured using IHC staining using previously established protocols [15]. Rabbit polyclonal anti-B3GNT6 (21291-1-AP, Thermofisher, US) was used at a working dilution of 1:100 co. The scores were evaluated on the basis of staining intensity and percentage of positive cells for each section. The staining intensity was scored as follows: 0, no staining; 1, light yellow staining; 2, yellow-brown staining; and 3, deep brown staining. The percentage of positive cells was scored as follows: 0, 0~5%; 1, 6~25%; 2, 26~50%; 3, 51~75%; and 4, > 75%. The final score was calculated using the following formula: positive cell score × staining intensity score. The total scores were condensed into four categories: 0 for negative (−), 1–3 for weakly positive (+), 4–7 for positive (++), and 8–12 for strongly positive (+++). All patients were sorted into two groups according to the total scores. High expression of B3GNT6 protein was defined as a detectable immunoreaction with a total score of ≥1+.

### Statistical analysis

Statistical analyses were performed using R studio (version 1.3.1056) and Graphpad Prism (Version 8.0.2). The comparison between B3GNT6 mRNA expression levels in colorectal cancer and normal tissues from TCGA and GEO databases was performed using unpaired Student’s *t* tests. The diagnostic value of B3GNT6 mRNA expression was evaluated using a receiver operating characteristic (ROC) curve. Survival analysis was conducted using log-rank (Mantel-Cox) test. The association between clinicopathological characteristics and B3GNT6 mRNA expression levels was determined using the *χ*^2^ test. p < 0.05 was considered statistically significant.

## Results

### B3GNT6 mRNA expression is downregulated in colorectal cancer tissues

**Fig. 1:**
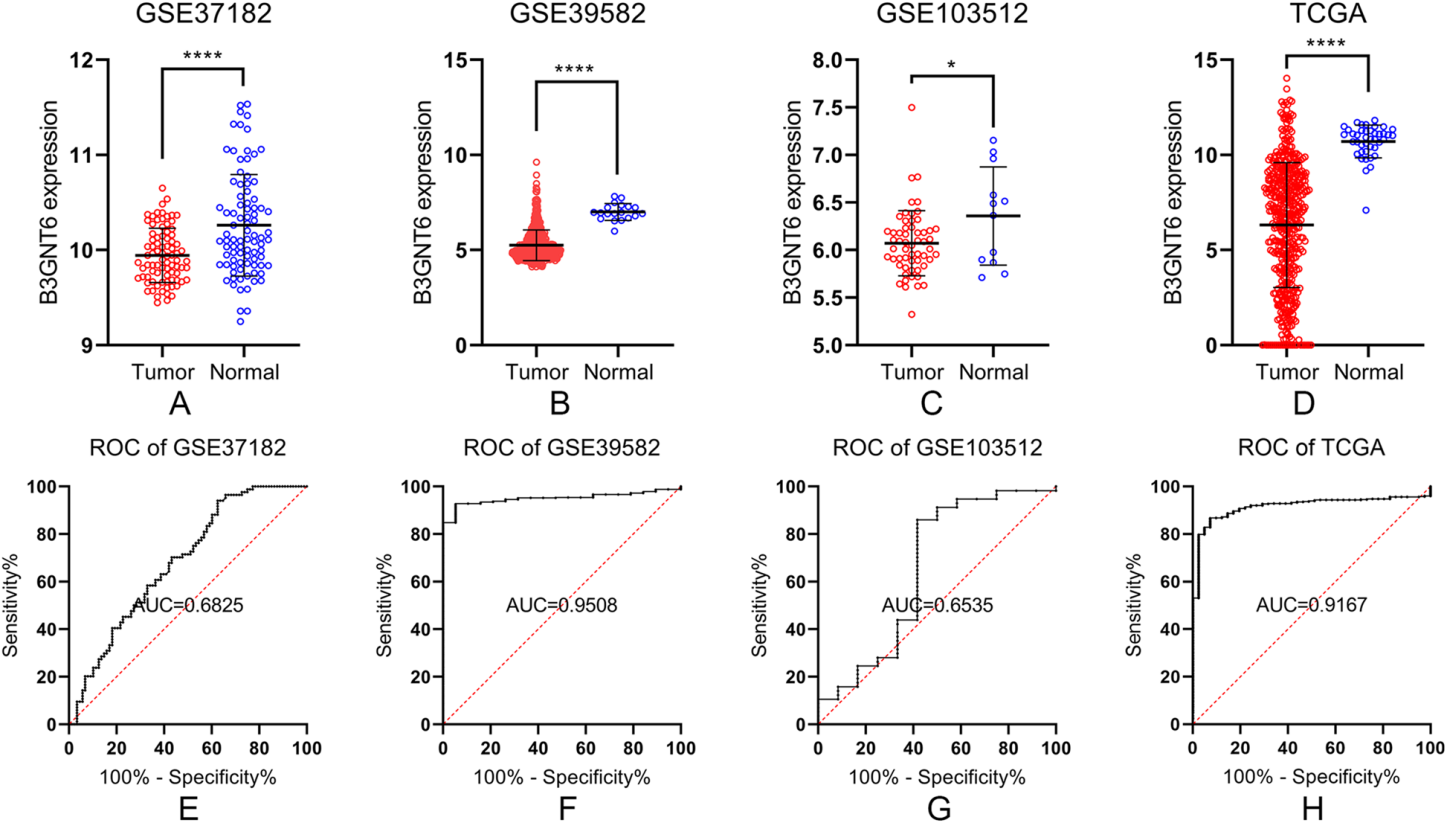
Bioinformatics analysis of B3GNT6 mRNA levels through online databases. **A**–**D** B3GNT6 mRNA levels in colorectal cancer tissues in comparison with those in normal tissues from GEO datasets (GSE37182, GSE39582, and GSE103512) and TCGA database. **E**–**H** The diagnostic value of B3GNT6 mRNA levels in online datasets

To measure B3GNT6 mRNA levels in colorectal cancer tissues, we first analyzed B3GNT6 mRNA levels by comparing mRNA levels in the tumor tissues compared with that in normal or para-tumor tissues in GSE37182, GSE39582, and GSE103512. B3GNT6 mRNA levels were significantly lower in tumor tissues than those in normal tissues in all three microarrays. This was further validated using the TCGA online database (Fig. 1A–D). The diagnostic value of B3GNT6 mRNA was measured by an ROC curve. Data from TCGA, GSE37182, and GSE39582 datasets revealed that B3GNT6 expression could be used as a good diagnostic marker for colorectal cancer with statistical significance (Fig. 1E–H). The area under the curve (AUC) of the four ROC curves was all greater than 0.6, demonstrating a good diagnostic value. The GSE39582 dataset showed the highest AUC (0.9508); when the cut-off value was <6.533, the sensitivity, specificity, positive predictive value (PPV), and negative predictive value (NPV) were 92.76%, 94.74%, 94.6%, and 93%, respectively. The accuracy was the highest, at 93.75%. To analyze B3GNT6 mRNA levels in other organs, we used the IST Online database (http://ist.medisapiens.com/). Results indicated that in the GI tract, the stomach, esophagus, and normal colorectal tissues, B3GNT6 mRNA levels were higher than those in colorectal cancer tissues; however, the small intestine showed low B3GNT6 expression. B3GNT6 mRNA levels were also higher in the bronchus (Supplementary Fig. 1).

### Correlation between B3GNT6 mRNA levels and clinicopathological characteristics in the GSE39582 dataset

We then focused on the GSE39582 dataset, which had the largest sample size and the most detailed information (Table 1). The dataset comprised two independent cohorts, the testing cohort (*n* = 443) and validating cohort (*n* = 123). Subgroup analysis showed that the chromosome instability (CIN) and KRAS mutated groups had patients with lower B3GNT6 mRNA levels.

**Table 1:** Association between B3GNT6 level and clinicopathological factors from dataset GSE39582

Variables	Testing cohort (*n*=443)			Validating cohort (*n*=123)		
	High (*n*=170)	Low (*n*=273)	*p* value	High (*n*=49)	Low (*n*=74)	*p* value
Age						
≥65	101	166	0.7351	35	52	0.8901
<65	69	106		14	22	
Unavailable	0	1		0	0	
Gender						
Male	90	147	0.8527	32	41	0.2738
Female	80	126		17	33	
Stage						
I-II	84	137	0.9321	28	48	0.3882
III-IV	82	136		21	26	
T stage						
T1-T2	21	26	0.3574	2	11	0.0625
T3-T4	145	239		43	59	
Unavailable	4	8		4	4	
Lymph node metastasis						
Absent	88	142	0.8803	26	46	0.3906
Present	76	119		19	24	
Unavailable	6	12		4	4	
Distant metastasis						
Absent	150	226	0.1474	40	66	0.3078
Present	16	38		4	3	
Unavailable	4	9		5	5	
Tumor Location						
Distal	96	171	0.1971	27	48	0.2772
Proximal	74	102		22	26	
MMR						
dMMR	18	43	0.1222	5	9	0.6202
pMMR	139	209		41	55	
Unavailable	13	21		3	10	
CIMP status						
−	111	195	0.1855	38	61	0.4994
+	33	41		8	9	
Unavailable	26	37		3	4	
CIN status						
negative	41	41	*0.0205*	17	11	*0.0059*
positive	98	176		25	55	
Unavailable	31	56		7	8	
tp53 mutation						
Mutated	51	84	0.9483	20	35	0.2642
Wild type	42	68		24	27	
Unavailable	77	121		5	12	
Kras mutation						
Mutated	84	88	*0.0002*	20	25	0.496
Wild type	78	174		29	47	
Unavailable	8	11		0	2	
Braf mutation						
Mutated	19	25	0.4532	4	3	0.3401
Wild type	130	218		44	69	
Unavailable	21	30		1	2	

*MMR* mismatch repair, *dMMR* mismatch repair-deficient, *pMMR* mismatch repair-proficient, *CIMP* CpG island methylator phenotype, *CIN* chromosome instability

### B3GNT6 protein expression is downregulated in colorectal cancer tissues

**Fig. 2:**
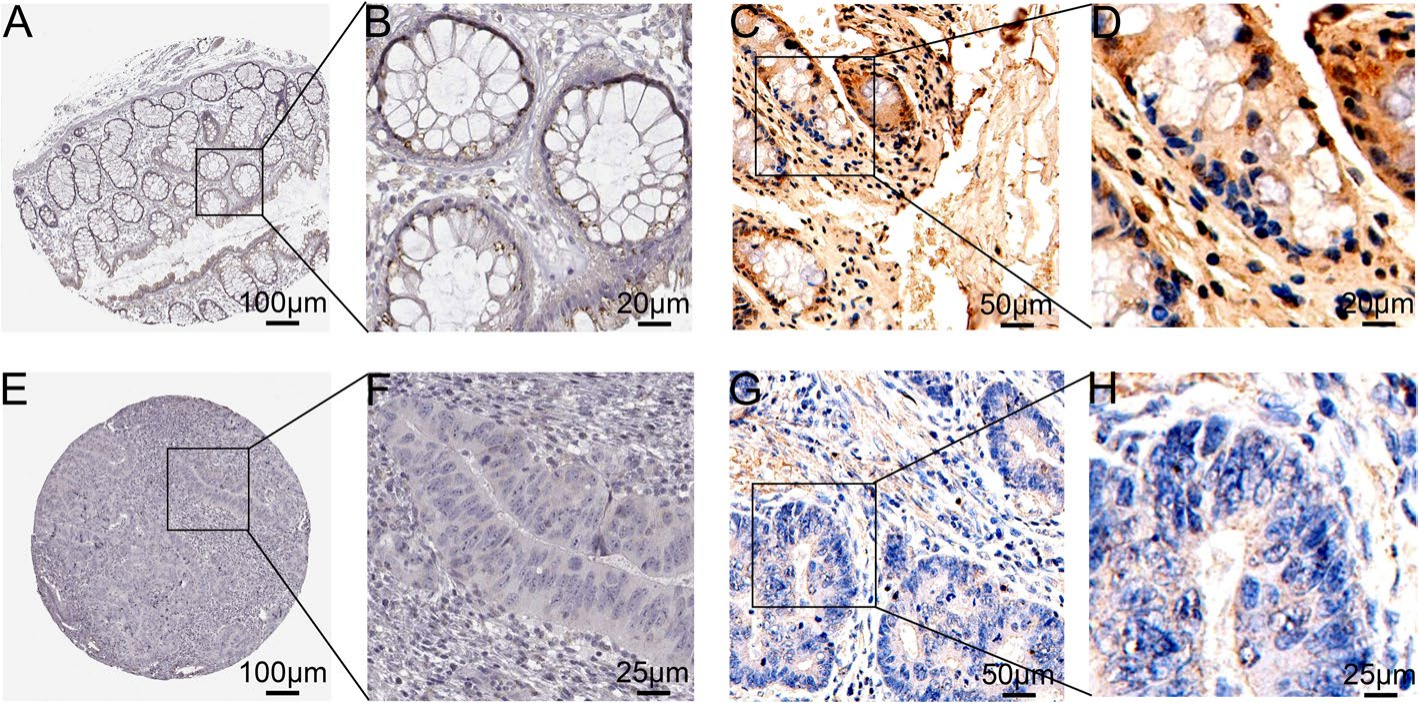
B3GNT6 protein expression levels were lower in colorectal cancer tissues than those in the normal tissue. **A**–**B**, **E**–**F** Immunohistochemical staining of B3GNT6 protein in normal colon and colorectal cancer tissues from the online database Human Protein Atlas. **C**–**D**, **G**–**H** Immunohistochemical staining of B3GNT6 protein in the normal colon and colorectal cancer tissues from our own clinical cohort. Corresponding scale bars are shown in (**A**–**H**) as 100 μm, 20 μm, 50 μm, 20 μm, 100 μm, 25 μm, 50 μm, and 25 μm

**Fig. 3:**
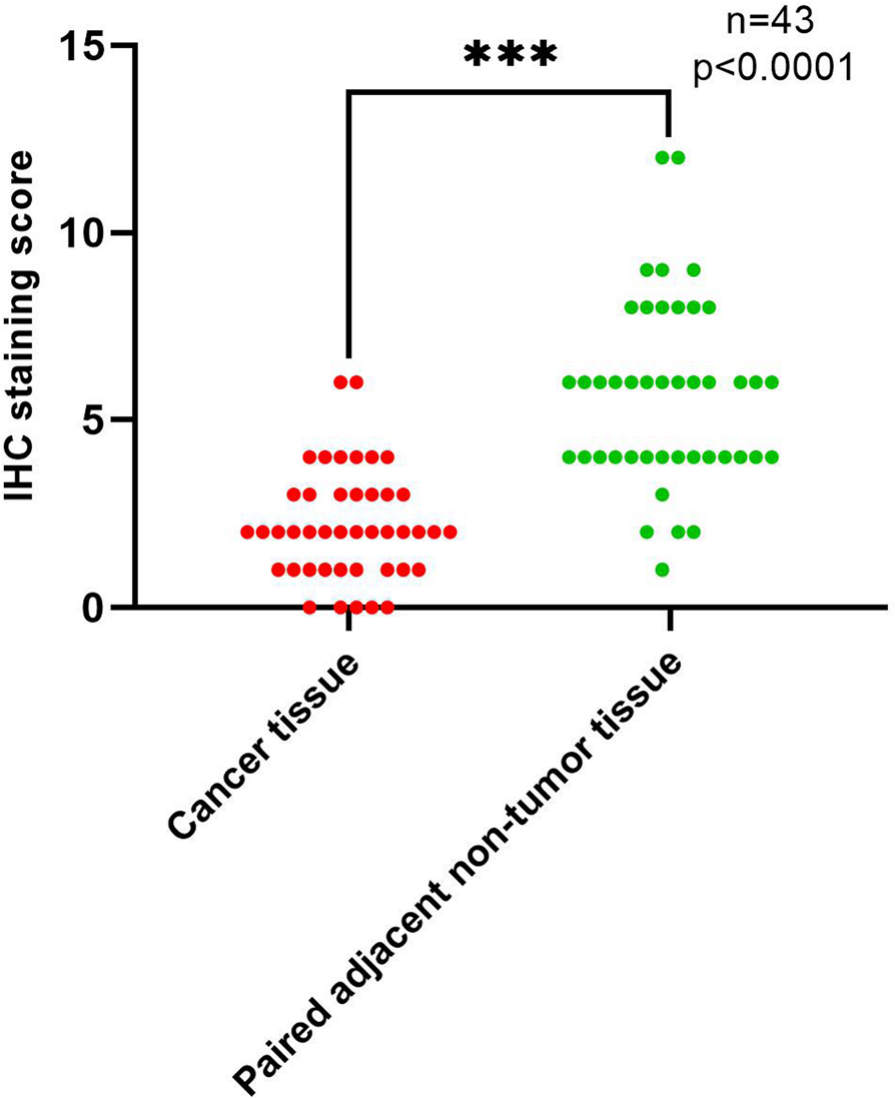
Immunohistochemical staining intensity score of colorectal cancer tissues and paired adjacent non-tumor tissues

To further address the change in B3GNT6 expression and its clinical significance in patients with colorectal cancer, we analyzed the online databases of protein expression together with our own clinical cohorts. We downloaded and analyzed B3GNT6 immunohistochemical micrographs in colorectal cancer and normal colon tissues from the Human Protein Atlas (https://www.proteinatlas.org/). Negative (11/12) or low (1/12) staining of B3GNT6 protein was observed in colorectal cancer tissues when compared to the medium staining in normal colon (3/3) and rectal tissues (3/3) (Fig. 2A, B, E, and F). The staining of B3GNT6 protein was largely localized to the cytoplasmic area, supposably in the Golgi apparatus area [10]. We also conducted immunohistochemical analyses in 43 colorectal cancer tissues with paired adjacent non-tumor tissues, which also showed downregulation of B3GNT6 protein expression in colorectal cancer. Negative (5/43) or weakly positive (30/43) or positive (8/43) staining of B3GNT6 protein was observed in colorectal cancer tissues when compared to the weakly positive (5/43) or positive (27/43) staining or strongly positive (11/43) staining in paired adjacent non-tumor tissues (Fig. 2C, D, G, and H and Fig. 3).

### Upregulation of B3GNT6 expression is associated with better overall survival in patients with colorectal cancer

**Fig. 4:**
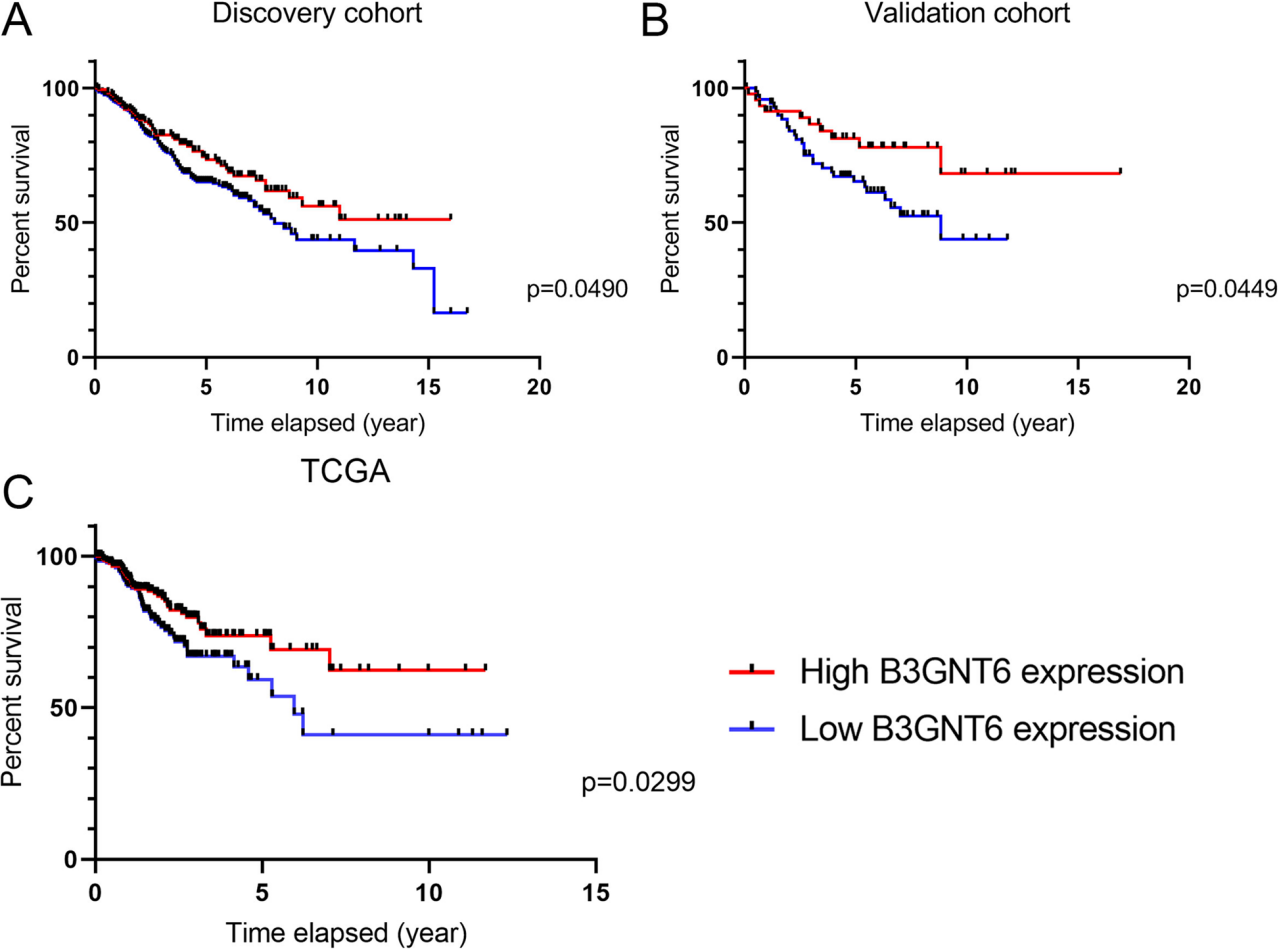
Increased B3GNT6 expression predicts better overall survival in patients with colorectal cancer. **A**, **B** Both the discovery and validation cohorts in GSE39582 comprised patients with high B3GNT6 mRNA levels showed better overall survival. **C** This was further validated using clinical data from TCGA online database

Next, we examined B3GNT6 expression with regard to its prognostic significance in colorectal cancer. In the GSE39582 dataset, patients with high B3GNT6 levels showed better overall survival than those with low B3GNT6 levels. This was further validated using the TCGA dataset (Fig. 4A–C). Together, these findings indicate that high B3GNT6 expression has clinical significance and could potentially serve as an important biomarker for predicting clinical outcomes in patients with colorectal cancer.

### Downregulation of B3GNT6 expression is correlated with upregulated proteasome activity

**Fig. 5:**
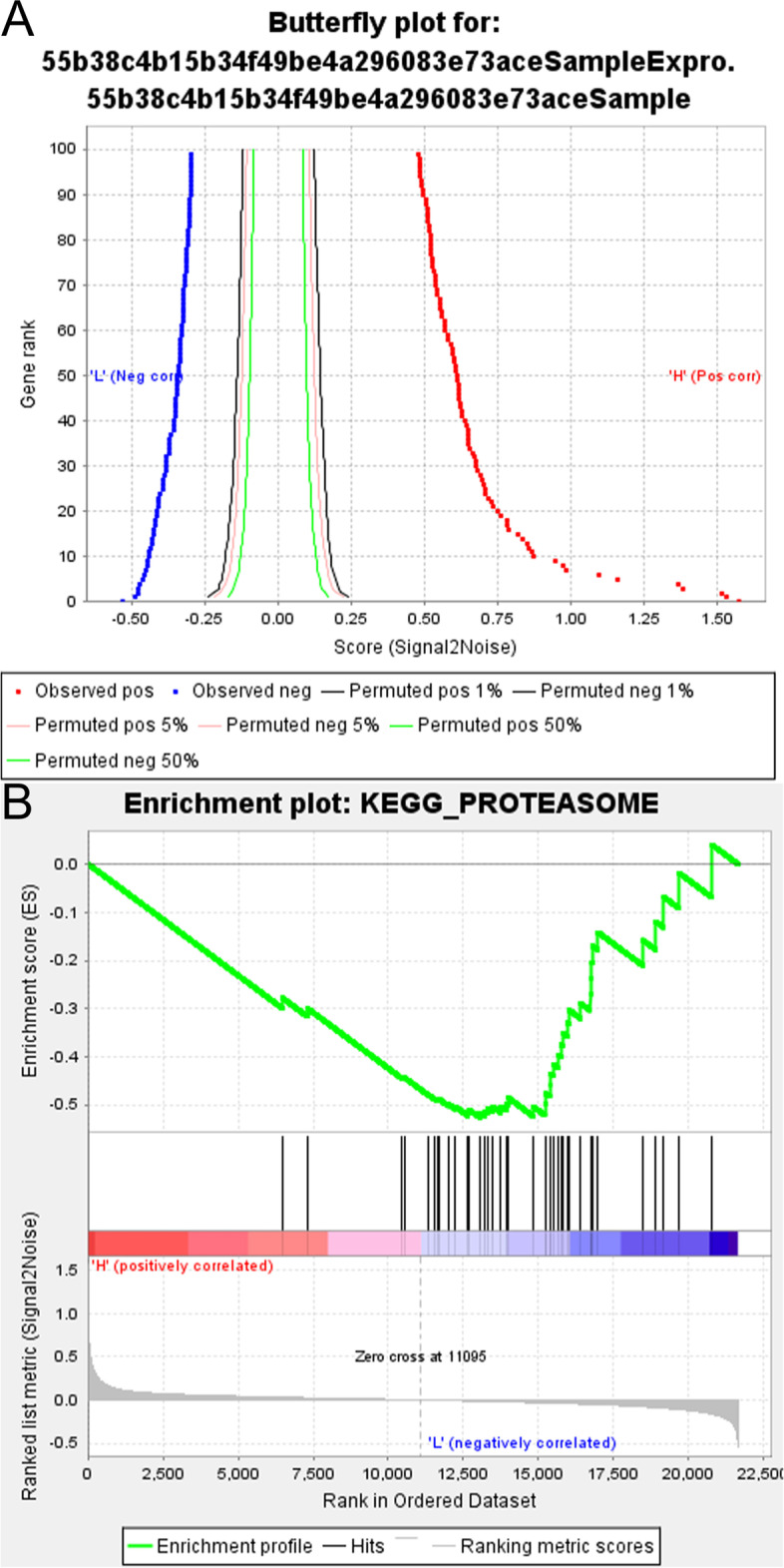
GSEA indicates low B3GNT6 levels are associated with increased proteasome activity. **A** The butterfly plot shows the positive and negative correlation between gene rank and the ranking metric score. **B** The enrichment plot indicates that B3GNT6 levels are negatively correlated with proteasome activity in the KEGG pathway

Based on the abovementioned analysis, it is likely that B3GNT6 might act as a tumor suppressor in the colorectal cancer microenvironment. To better understand the mechanism underlying the role of B3GNT6 as a tumor suppressor, gene set enrichment analysis (GSEA) was conducted in the GSE39582 dataset. The top 25% and bottom 25% B3GNT6 mRNA levels in patients enrolled in this study were used for GSEA. We adopted both KEGG and Biocarta pathway analyses in GSEA, and both pathway analyses indicated that B3GNT6 mRNA levels were negatively correlated with the ubiquitin-proteasome system (Table 2, Fig. 5, and Supplementary Fig. 2).

**Table 2:** GSEA analysis of B3GNT6 mRNA expression in GSE39582

Geneset name	NES	NOM *p*-val	FDR *q*-val
BIOCARTA pathway			
Upregulated			
BIOCARTA_RAB_PATHWAY	1.6703635	0.026	0.33836955
BIOCARTA_CERAMIDE_PATHWAY	1.6385397	0.03807615	0.34184185
Downregulated			
*BIOCARTA_PROTEASOME_PATHWAY*	−*1.865893*	*0.011235955*	*0.15191999*
KEGG pathway			
Upregulated			
KEGG_OLFACTORY_TRANSDUCTION	2.0274692	0	0.027611418
KEGG_TASTE_TRANSDUCTION	1.7740936	0	0.19784024
KEGG_GLYCOSPHINGOLIPID_BIOSYNTHESIS_LACTO_AND_NEOLACTO_SERIES	1.770417	0.001926782	0.13674085
KEGG_O_GLYCAN_BIOSYNTHESIS	1.7251008	0.007736944	0.16378903
KEGG_GNRH_SIGNALING_PATHWAY	1.7178557	0	0.14160849
KEGG_ALZHEIMERS_DISEASE	1.6913323	0.026923077	0.15125382
KEGG_NITROGEN_METABOLISM	1.6708751	0.001992032	0.13392551
KEGG_LONG_TERM_POTENTIATION	1.64782	0.024528302	0.14832915
KEGG_HUNTINGTONS_DISEASE	1.638019	0.04660194	0.14545798
KEGG_VASOPRESSIN_REGULATED_WATER_REABSORPTION	1.63744	0.018072288	0.1325422
KEGG_BUTANOATE_METABOLISM	1.6251645	0.01778656	0.13560429
KEGG_DRUG_METABOLISM_OTHER_ENZYMES	1.6193944	0.01178782	0.13172121
KEGG_PORPHYRIN_AND_CHLOROPHYLL_METABOLISM	1.5756354	0.025742574	0.17885368
KEGG_MATURITY_ONSET_DIABETES_OF_THE_YOUNG	1.5706768	0.027613413	0.17375107
KEGG_TERPENOID_BACKBONE_BIOSYNTHESIS	1.5477382	0.046092186	0.19446045
KEGG_AMYOTROPHIC_LATERAL_SCLEROSIS_ALS	1.5473746	0.023391813	0.18385722
KEGG_GLYCEROPHOSPHOLIPID_METABOLISM	1.5310374	0.015873017	0.1866673
KEGG_RETINOL_METABOLISM	1.526893	0.02970297	0.18341947
KEGG_GLYCOLYSIS_GLUCONEOGENESIS	1.5237751	0.038	0.17888556
KEGG_VIBRIO_CHOLERAE_INFECTION	1.5188705	0.020876827	0.17640822
KEGG_PROXIMAL_TUBULE_BICARBONATE_RECLAMATION	1.4940959	0.048543688	0.20143263
KEGG_STARCH_AND_SUCROSE_METABOLISM	1.4879901	0.037848607	0.20171466
KEGG_ASCORBATE_AND_ALDARATE_METABOLISM	1.4647686	0.03976143	0.21592942
Downregulated			
KEGG_BASAL_TRANSCRIPTION_FACTORS	−1.7745181	0.009920635	0.3991878
*KEGG_UBIQUITIN_MEDIATED_PROTEOLYSIS*	−*1.773844*	*0.013861386*	*0.20233367*
KEGG_RNA_DEGRADATION	−1.6950728	0.028688524	0.21618877
KEGG_GLYCOSAMINOGLYCAN_BIOSYNTHESIS_HEPARAN_SULFATE	−1.5696611	0.020715632	0.3765442
KEGG_GLYCOSAMINOGLYCAN_BIOSYNTHESIS_CHONDROITIN_SULFATE	−1.5518181	0.04183267	0.3753312

*NES* normalized enrichment score, that is, the enrichment score for the gene set after it has been normalized across analyzed gene sets. *FDR q-val* false discovery rate, that is, the estimated probability that the normalized enrichment score represents a false positive finding. *NOM p-val* normalized p value, that is, the statistical significance of the enrichment score. The nominal p value is not adjusted for gene set size or multiple hypothesis testing; therefore, it is of limited use in comparing gene sets

## Discussion

We conducted a bioinformatic analysis of GEO and TCGA datasets as well as our own cohort and found that the mRNA and protein levels of B3GNT6 in colorectal cancer tissues were significantly lower than those in the normal tissues, which was consistent with the results of previous studies [10]. Survival analysis showed that the downregulation of B3GNT6 mRNA expression was associated with a low overall survival rate of patients with colorectal cancer. The downregulation of B3GNT6 mRNA expression in colorectal cancer tissues is related to CIN status, KRAS mutation, and proteasome pathway.

Our study found that colorectal cancer patients with low B3GNT6 levels are more likely to develop KRAS mutations and chromosomal instability (CIN). This is interesting because it may clarify the role of B3GNT6 expression in the development of colorectal cancer. The chromosomal instability (CIN) pathway is usually driven by sequence mutation events, the most common being KRAS, which leads to typical aneuploid microsatellite stable (MSS) colorectal cancer, and the sequence mutation event that drives CIN is more likely to be KRAS than BRAF [16]. The results of bioinformatics analyses showed that the levels of B3GNT6 mRNA expression in the KRAS mutation group were lower. We speculated that B3GNT6 might negatively regulate the occurrence and metastasis of colorectal cancer through the KRAS/ERK signal pathway. Previous research supports this hypothesis that the KRAS/ERK signaling pathway is closely related to OGT levels in pancreatic, gastric, and cervical cancers [17–19].

The B3GNT6 protein is a key enzyme in the synthesis of the core 3 structure of O-glycans, and its low expression or non-expression causes aberrant protein O-GlcNAcylation. A study by Radhakrishnan et al. revealed that the expression of the core 3 structure of O-glycans affects the production and stability regulation of α1 and β2 integrin in pancreatic cancer, thus inhibiting metastasis [6]. Boottanun and colleagues reported that the low expression of B3GNT6 is associated with higher differentiation and better prognosis in patients with cholangiocarcinoma [7]. In prostate cancer, B3GNT6 inhibits tumor formation and metastasis through the downregulation of the α2-β1 integrin complex [9]. Li et al. reported that α1 integrin enhanced tumorigenicity and invasiveness of colorectal cancer. It is worth noting that this mechanism acts through the activation of the KRAS/ERK signaling pathway [8]. This may confirm our hypothesis that the B3GNT6 protein regulates the KRAS/ERK signaling pathway. Further molecular biological studies are warranted to validate the regulatory effect of B3GNT6 expression on the KRAS signaling pathway.

Our results also showed that the downregulation of B3GNT6 mRNA expression levels is related to increased proteasome activity. This is interesting because proteasomes often form a proteasome system (UPS) with ubiquitin. Under normal physiological conditions, the UPS is responsible for eliminating dysfunctional/misfolded proteins through the proteasome. These specific functions enable the UPS to regulate protein quality in cells, thus maintaining cellular homeostasis and cell survival [20]. Dysregulation of the UPS is commonly observed in various cancers, and its dysfunction may promote tumor development by regulating the degradation of specific proteins [21, 22]. Over the past decade, the United States Food and Drug Administration has approved a few UPS inhibitors for hematological malignant tumors and these drugs have already achieved curative effects in a few cases. However, due to the characteristics of the proteasome itself, the development of drugs based on protein degradation is rather slow. Presently, the practice of UPS inhibitors in the field of colorectal cancer and other solid tumors is still in the exploratory stage, and the limited clinical remission rate makes it not put into clinical application on a large scale for the time being [23–25].

Our understanding of the specific mechanism of UPS remains limited, but given the results of previous studies and ours [22, 25], we consider that the increased activity of UPS promotes the occurrence and metastasis of colorectal cancer and eventually leads to a poor prognosis for patients. In tumor environments, downregulation of B3GNT6 expression may lead to upregulation of proteasome activity, which in turn suppresses the accumulation of misfolded or toxic proteins in tumor cells. This disrupts the apoptosis of these tumor cells that eventually results in tumor formation. Further well-designed studies focusing on the role of B3GNT6 in proteasome regulation are necessary because their association has not been reported as yet.

## Conclusions

The findings of this study demonstrate that low B3GNT6 expression is a potential biomarker of poor outcomes in patients with CRC and may lead to more frequent activation of the KRAS/ERK signaling pathway, CIN, and increased proteasomal activity. These new results may prove helpful in the molecular diagnosis of CRC as well as the creation of a new therapeutic target for CRC. Further confirmation using a large and multi-center clinical cohort is required for the validation of its predictive ability. Further laboratory diagnosis should be undertaken to verify the molecular mechanism underlying the relationship between B3GNT6 levels and KRAS mutation, CIN, and increased proteasomal activity.

## Supplementary Information

**Additional file 1: Supplementary Figure 1**. B3GNT6 mRNA levels in healthy tissues (green) compared with cancer tissues (red), downloaded from IST online database (http://ist.medisapiens.com/).

**Additional file 2: Supplementary Figure 2**. The heat map shows patients with high B3GNT6 level (above) and low B3GNT6 level (below) and their gene expression in proteasome pathway.

## Data Availability

The datasets generated and/or analyzed during the current study are available in the Gene Expression Omnibus (GEO) database (https://www.ncbi.nlm.nih.gov/geo/), TCGA dataset (https://www.portal.gdc.cancer.gov/), MediSapiens IST Online database (http://ist.medisapiens.com/), and the Human Protein Atlas (http://www.proteinatlas.org/).

## Abbreviations

CIN: Chromosome instability
MIN: Microsatellite instability
CIMP: CpG island methylator phenotype
MSS: Microsatellite stable
B3GNT6: UDP-GlcNAc:BetaGal Beta-1,3-*N*-acetylglucosaminyltransferase 6
GEO: Gene Expression Omnibus
TCGA: The Cancer Genome Atlas
UPS: Ubiquitin-proteasome system
ROC: Receiver operating characteristic

## References

[1] Dekker E, Tanis PJ, Vleugels JLA, Kasi PM, Wallace MB (2019). Colorectal cancer. Lancet (London, England).

[2] Brenner H, Kloor M, Pox CP (2014). Colorectal cancer. Lancet (London, England).

[3] Dienstmann R, Vermeulen L, Guinney J, Kopetz S, Tejpar S, Tabernero J (2017). Consensus molecular subtypes and the evolution of precision medicine in colorectal cancer. Nat Rev Cancer.

[4] Brockhausen I (1999). Pathways of O-glycan biosynthesis in cancer cells. Biochim Biophys Acta.

[5] Kim YS (1998). Mucin glycoproteins in colonic neoplasia. Keio J Med.

[6] Radhakrishnan P, Grandgenett PM, Mohr AM, Bunt SK, Yu F, Chowdhury S (2013). Expression of core 3 synthase in human pancreatic cancer cells suppresses tumor growth and metastasis. Int J Cancer.

[7] Boottanun P, Ino Y, Shimada K, Hiraoka N, Angata K, Narimatsu H (2021). Association between the expression of core 3 synthase and survival outcomes of patients with cholangiocarcinoma. Oncol Lett.

[8] Li H, Wang Y, Rong S-K, Li L, Chen T, Fan Y-Y (2020). Integrin α1 promotes tumorigenicity and progressive capacity of colorectal cancer. Int J Biol Sci.

[9] Lee SH, Hatakeyama S, Yu SY, Bao X, Ohyama C, Khoo KH (2009). Core3 O-glycan synthase suppresses tumor formation and metastasis of prostate carcinoma PC3 and LNCaP cells through down-regulation of alpha2beta1 integrin complex. J Biol Chem.

[10] Iwai T, Kudo T, Kawamoto R, Kubota T, Togayachi A, Hiruma T (2005). Core 3 synthase is down-regulated in colon carcinoma and profoundly suppresses the metastatic potential of carcinoma cells. Proc Natl Acad Sci U S A.

[11] Wolf DH, Hilt W (2004). The proteasome: a proteolytic nanomachine of cell regulation and waste disposal. Biochim Biophys Acta.

[12] Marisa L, de Reyniès A, Duval A, Selves J, Gaub MP, Vescovo L (2013). Gene expression classification of colon cancer into molecular subtypes: characterization, validation, and prognostic value. PLoS Med.

[13] Brouwer-Visser J, Cheng WY, Bauer-Mehren A, Maisel D, Lechner K, Andersson E (2018). Regulatory T-cell genes drive altered immune microenvironment in adult solid cancers and allow for immune contextual patient subtyping. Cancer Epidemiol Biomarkers Prev.

[14] Musella V, Verderio P, Reid JF, Pizzamiglio S, Gariboldi M, Callari M (2013). Effects of warm ischemic time on gene expression profiling in colorectal cancer tissues and normal mucosa. PLoS One.

[15] Wang X, Yu Q, Ghareeb WM, Zhang Y, Lu X, Huang Y (2019). Downregulated SPINK4 is associated with poor survival in colorectal cancer. BMC Cancer.

[16] Menter DG, Davis JS, Broom BM, Overman MJ, Morris J, Kopetz S (2019). Back to the colorectal cancer consensus molecular subtype future. Curr Gastroenterol Rep.

[17] Qian K, Wang S, Fu M, Zhou J, Singh JP, Li M-D (2018). Transcriptional regulation of *O*-GlcNAc homeostasis is disrupted in pancreatic cancer. J Biol Chem.

[18] Jiang M, Qiu Z, Zhang S, Fan X, Cai X, Xu B (2016). Elevated O-GlcNAcylation promotes gastric cancer cells proliferation by modulating cell cycle related proteins and ERK 1/2 signaling. Oncotarget..

[19] Wu H, Song S, Yan A, Guo X, Chang L, Xu L (2020). RACK1 promotes the invasive activities and lymph node metastasis of cervical cancer via galectin-1. Cancer Lett.

[20] Collins GA, Goldberg AL (2017). The logic of the 26S proteasome. Cell..

[21] Manasanch EE, Orlowski RZ (2017). Proteasome inhibitors in cancer therapy. Nat Rev Clin Oncol.

[22] Tokheim C, Wang X, Timms RT, Zhang B, Mena EL, Wang B (2021). Systematic characterization of mutations altering protein degradation in human cancers. Mol Cell.

[23] Soave CL, Guerin T, Liu J, Dou QP (2017). Targeting the ubiquitin-proteasome system for cancer treatment: discovering novel inhibitors from nature and drug repurposing. Cancer Metastasis Rev.

[24] Milacic V, Chen D, Ronconi L, Landis-Piwowar KR, Fregona D, Dou QP (2006). A novel anticancer gold (III) dithiocarbamate compound inhibits the activity of a purified 20S proteasome and 26S proteasome in human breast cancer cell cultures and xenografts. Cancer Res.

[25] Konstantinopoulos PA, Papavassiliou AG (2006). The potential of proteasome inhibition in the treatment of colon cancer. Expert Opin Investig Drugs.

