# Supplementary figures and images for: Downregulation of B3GNT6 is a predictor of poor outcomes in patients with colorectal cancer
Source: World J Surg Oncol. 2022 Apr 7;20:110. doi: 10.1186/s12957-022-02561-x (PMC8988341; doi:10.1186/s12957-022-02561-x)

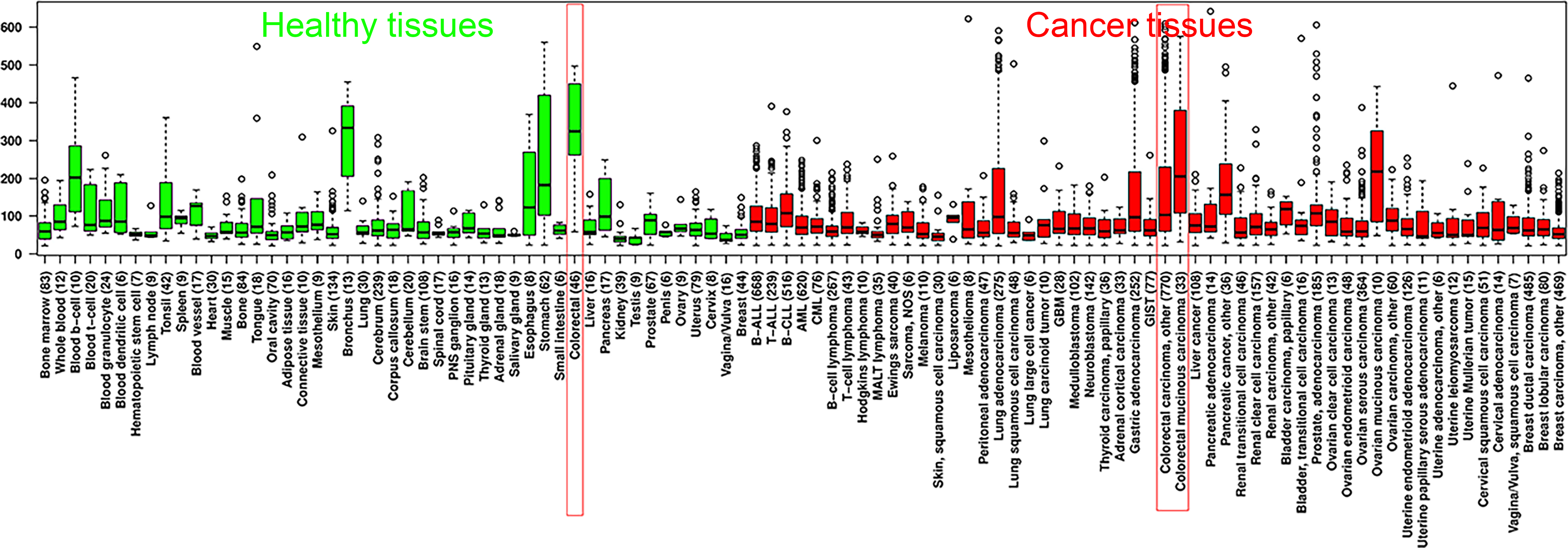

Supplement: Supplementary file 1 — Additional file 1: Supplementary Figure 1. B3GNT6 mRNA levels in healthy tissues (green) compared with cancer tissues (red), downloaded from IST online database (http://ist.medisapiens.com/). [file 12957_2022_2561_MOESM1_ESM.tif]
